# Visuomotor Correction is a Robust Contributor to Force Variability During Index Finger Abduction by Older Adults

**DOI:** 10.3389/fnagi.2015.00229

**Published:** 2015-12-15

**Authors:** Brian L. Tracy, Leah N. Hitchcock, Seth J. Welsh, Roger J. Paxton, Caitlin E. Feldman-Kothe

**Affiliations:** Department of Health and Exercise Science, Colorado State UniversityFort Collins, CO, USA

**Keywords:** steadiness, force fluctuations, aging, elderly, visual processing, motor variability, hand, dexterity

## Abstract

We examined aging-related differences in the contribution of visuomotor correction to force fluctuations during index finger abduction via the analysis of two datasets from similar subjects. Study (1) Young (*N* = 27, 23 ± 8 years) and older adults (*N* = 14, 72 ± 9 years) underwent assessment of maximum voluntary contraction force (MVC) and force steadiness during constant-force (CF) index finger abduction (2.5, 30, 65% MVC). For each trial, visual feedback of the force (VIS) was provided for 8–10 s and removed for 8–10 s (NOVIS). Visual gain of the force feedback at 2.5% MVC was high; 12- and 26-fold greater than the 30 and 65% MVC targets. Mean force, standard deviation (SD) of force, and coefficient of variation (CV) of force was calculated for detrended (<0.5 Hz drift removed) VIS and NOVIS data segments. Study (2) A similar group of 14 older adults performed discrete, randomly-ordered VIS or NOVIS trials at low target forces (1–3% MVC) and high visual gain. Study (1) For young adults the CV of force was similar between VIS and NOVIS for the 2.5% (4.8 vs. 4.3%), 30% (3.2 vs. 3.2%) and 65% (3.5 vs. 4.2%) target forces. In contrast, for older adults the CV of force was greater for VIS than NOVIS for 2.5% MVC (6.6 vs. 4.2%, *p* < 0.001), but not for the 30% (2.4 vs. 2.4%) and 65% (3.1 vs. 3.3%) target forces. At 2.5% MVC, the increase in CV of force for VIS compared with NOVIS was significantly greater (age × visual condition *p* = 0.008) for older than young adults. Study (2) Similarly, for older adults performing discrete, randomly ordered trials the CV of force was greater for VIS than NOVIS (6.04 vs. 3.81%, *p* = 0.01). When visual force feedback was a dominant source of information at low forces, normalized force variability was ~58% greater for older adults, but only 11% greater for young adults. The significant effect of visual feedback for older adults was not dependent on the order of presentation of visual conditions. The results indicate that impaired processing of visuomotor information underlies the greater motor variability observed in older adults during lab-based isometric contractions of a hand muscle.

## Introduction

The greater variability of force production and movement often exhibited by older adults is interesting from both a physiological and functional perspective (Enoka et al., 2003; Tracy, 2009). As adults age, something must be changing in the neuromuscular system to underlie the greater variability, thus many investigations have focused on possible central and peripheral physiological mechanisms (Galganski et al., 1993; Burnett et al., 2000; Laidlaw et al., 2000; Vaillancourt et al., 2001; Sosnoff and Newell, 2006b; Welsh et al., 2007). Degraded force control is also interesting from a functional perspective, due to the impact on successful daily function and mobility, a central feature of independance and quality of life for older adults (Cole, 1991; Kornatz et al., 2005; Seynnes et al., 2005; Marmon et al., 2011). The notion of a relation between force variability and function emanates from the theoretical realm (Hamilton and Wolpert, 2002) and from studies that showed an association between hand muscle force variability and manual dexterity (Marmon et al., 2011), a relation between changes in steadiness and changes in function (Kornatz et al., 2005), and greater variability in functionally impaired older adults (Seynnes et al., 2005; Carville et al., 2007).

The link between age-related impairment in force control and motor unit discharge variability has been reasonably well documented in isometric, concentric, and eccentric contractions (Laidlaw et al., 2000; Tracy and Enoka, 2002; Enoka et al., 2003; Tracy et al., 2007; Tracy, 2007a, 2009). The discharge behavior of motor units is often more variable in older adults (Laidlaw et al., 2000; Welsh et al., 2007; Jordan et al., 2013), suggesting altered properties of spinal motor neurons or altered synaptic input to motor neurons. For example, our group has observed greater variability of motor unit discharge during isometric contractions performed by older adults with high-gain visual feedback, compared with no visual feedback. This finding suggested that the greater variability of descending drive that occurs when older adults engage in visuomotor processing results in measurably greater fluctuations in the synaptic input to spinal motor neurons (Welsh et al., 2007).

Interestingly, the differences in isometric force variability between young and older adults are greater and more consistently found at the lowest target forces (Burnett et al., 2000; Laidlaw et al., 2000; Tracy et al., 2007). In many experiments that documented this age effect, the lowest target forces were displayed to the research subject with high visual gain, meaning that relatively small changes in force produced relatively large excursions of the force signal on the visual feedback monitor (Galganski et al., 1993; Burnett et al., 2000; Laidlaw et al., 2000; Tracy and Enoka, 2002; Taylor et al., 2003; Tracy et al., 2005b, 2007; Baweja et al., 2015). The gain of visual feedback can be a significant determinant of the variability of force for older adults (Sosnoff and Newell, 2006b; Tracy, 2009; Kennedy and Christou, 2011; Fox et al., 2013; Baweja et al., 2015). Accordingly, we performed studies in which young and older adults performed low force isometric contractions that were presented with either relatively high gain visual force feedback, or with no visual feedback (Tracy et al., 2007; Tracy, 2007b, 2009; Welsh et al., 2007; Paxton et al., 2015). In widely differing muscle groups (knee extensors, elbow flexors, ankle dorsiflexors, ankle plantarflexors), the older adults exhibited greater force variability compared with young adults only with visual feedback, with minimal age differences observed in the absence of visual feedback.

Those previous findings were primarily from larger muscles. Here, we conducted an age- and visual feedback-based comparison of force variability during isometric contractions of the first dorsal interosseus, an intrinsic hand muscle that is the single agonist for index finger abduction. There are substantial differences between larger muscles and small hand muscles in direct cortical input (Brouwer and Ashby, 1990; Dum and Strick, 1996), properties of the motor unit pool (Feinstein et al., 1955; Enoka and Fuglevand, 2001), afferent and interneuron input (Rossi and Mazzocchio, 1991; Katz et al., 1993; Durbaba et al., 2005), sensation of the limb (Stevens et al., 2003) and typical use (fine dexterity vs. power/locomotion). Therefore, it is of interest to determine whether the age-related visuomotor contribution to force fluctuations consistently found for large muscles also extends to small hand muscles. There is a long-documented impairment of information processing in aging humans (Wickens et al., 1987; Cerella and Hale, 1994). This impairment likely extends to the processing and integration of afferent information to produce a refined motor command (Sosnoff and Newell, 2006b). A finding of greater contribution of visuomotor correction for older adults, if found across large and small muscles with different neural properties and functional roles, would further strengthen the notion of impaired control of force in aging (greater force variability) as a central phenomenon associated with the processing of visuomotor information and correction of descending command. Two sets of findings led us to hypothesize that the force fluctuations would be greater for older than young adults during hand muscle contractions with high visual gain: (1) Recent findings from hand muscles on aging, visuomotor processing, and force variability indicating a role for impaired visuomotor processing (Kennedy and Christou, 2011; Baweja et al., 2015) and (2) our consistent demonstration, in older adults, of impaired visuomotor processing during contractions of large muscles. For consistency with other literature in this area, and to examine force control over a larger range of muscle activation, we also examined force fluctuations at two higher forces. In addition, analysis of data from a separately performed experiment on similar subjects allowed us to determine if the order of presentation of visual feedback conditions in previous standard protocols (vision then no-vision segments in single ~20 s trials) contributed to the greater fluctuations observed for older adults during vision conditions. Some of these data were presented previously in abstract form (Tracy et al., 2009).

## Materials and Methods

### Participants

Study 1: Twenty-seven young adults (22.7 ± 3.3 years, range: 18–30 years, 12 men, 15 women) and 14 older adult (71.9 ± 4.8 years, 66–81 years, 7 men, 7 women) subjects completed testing.

Study 2: Fourteen older adults (75.1 ± 5.02 years, 68–84 years, 5 men, 9 women) completed testing. The age for older adults in Study 2 was not different than the older adults from Study 1 (*p* > 0.05).

All subjects reported minimal regular exercise (<3 h per week of low to moderate intensity exercise, no strength training for at least the previous year) and no medications known to influence the measures. For older adults, a physician-supervised maximal treadmill test and a physical exam screened for overt neurological, cardiovascular, and other exclusionary health concerns. The young adults completed health history questionnaires for screening. In order to ensure that vision impairment did not affect the ability to utilize of visual feedback from the monitor, subjects viewed the target line and force line with their usual corrective lenses and were asked to confirm that they could clearly see and also understand the nature of the visual feedback. Subjects reported clearly seeing and understanding the visual feedback provided and understood the verbal commands given to them during testing. All subjects reported being right hand dominant. Volunteers were oriented to the protocol and provided written informed consent prior to participation. The experimental protocol was approved by the Human Research Committee at Colorado State University, which operates in accord with the Helsinki Declaration.

### Experimental Design

Participants completed the screening, orientation, consent, and experimental session in one two hour visit. Testing consisted of subjects exerting isometric abduction forces with the index finger of their non-dominant hand, during strength and force steadiness tasks.

### Experimental Setup

Participants were seated in an adjustable chair with straps on the hips and torso and the shoulder abducted approximately 20 degrees in zero degrees of flexion. The elbow joint was at a right angle and in line with the trunk. The elbow and forearm was enveloped and restrained by a vacuum pillow. The forearm was pronated with the hand palm down and 3rd–5th fingers comfortably immobilized. During testing the index finger was placed in a neutral position (5 degrees abduction) and strapped to a custom orthosis/load cell interface, with the thumb extended behind a rigid stop in the same plane as the hand, as in Laidlaw et al. (2000).

Load cells rated for either 222 N or 45 N maximum force (Transducer Techniques, Temecula, CA, USA) were used to measure abduction force perpendicular to the index finger and in line with the proximal interphalangeal joint. The testing position was chosen to best isolate the FDI muscle and minimize co-contraction of other muscles, based on pilot testing. Visual force feedback was provided on a 48 cm flat-panel LCD monitor placed 75 cm away from the subject. The screen displayed two bold horizontal lines; one that represented the force exerted by the subject and another that represented the target force. The subject’s force line moved up or down with increases or decreases in force. As done previously (Laidlaw et al., 2000; Tracy and Enoka, 2002; Tracy et al., 2007; Welsh et al., 2007), the visual feedback gain was adjusted so that the target force was always exactly mid-way up the screen. For Study 1, the gain of the visual feedback was therefore highest at the lowest target force (2.5% MVC) and declined proportionally at greater target forces (30%, 65% MVC). Thus, at the highest gain small changes in force were translated into relatively larger vertical excursions of the force line on the screen.

### Experimental Protocol

#### Maximal Voluntary Contraction (MVC) Task

Subjects performed 3–5 MVC trials with their FDI muscle. Trials were performed until the maximal forces from two trials were within 5% of each other. No subjects required more than five trials to complete this criterion. All subjects were given a practice trial prior to the test trials. A simple diagram guided the subject in proper task performance and visual feedback of their own force was provided for them during each trial. They were instructed to increase their index finger abduction force from rest to maximum over 3 s and push as hard as possible for 3 s with strong verbal encouragement. Sixty seconds of rest was given between MVC trials.

#### Constant-Force Task

**Figure 1:**
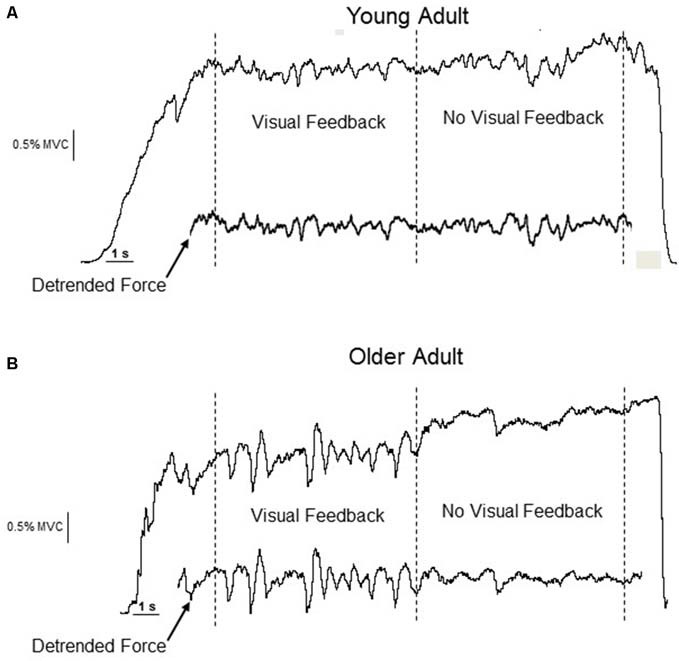
**Experimental data from a young (A) and older (B) adult performing an isometric constant-force task at 2.5% MVC.** The visual feedback segment precedes the no-visual feedback segment. The detrended force (drift < 0.5 Hz removed) is inset below the original force data. The removal of drift, preservation of the fluctuations in the detrended force trace, and the qualitative change in the amplitude of the force fluctuations for the older adult is evident.

Study 1: After a practice trial, two test trials were completed at each of three submaximal target force levels (2.5, 30, 65% MVC). Target forces were presented in random order. Subjects were instructed to increase their FDI abduction force up to the target line over two seconds and hold it as steady as possible at the target force (~20 s). Before each trial, subjects were informed that midway during the trial the screen would be turned off and they were instructed to continue holding the same force as steadily as possible (Figure 1). Visual feedback of the force was thus provided for approximately 10 s (VIS) and then removed for approximately 10 s (NOVIS) during each trial (Tracy et al., 2007). A minimum of 30 s of rest was given to subjects after each 2.5% MVC and 30% MVC trial and 60 s after 65% MVC trials, to minimize the risk of fatigue (none reported) at higher force levels.

Study 2: Subjects completed constant-force trials with and without visual feedback of the force, but the trials for each visual condition were performed separately from each other and the order of presentation was randomized. For no-vision trials the subjects were allowed visual feedback to get up to the target force and then the visual feedback was immediately removed. For the discrete trials the target force averaged 2.0% MVC (1.02–2.9% MVC), and the target line was always placed midway up the screen, thus the visual gain was relatively high and similar to the trials performed for Study 1. Rather than a nominal 2.5% MVC target force, the target forces were within this low 1–3% MVC range because single motor unit discharge was being recorded at just above recruitment threshold (data not presented). Subjects were instructed to hold the force at the target as steadily as possible for VIS and NOVIS.

### Data Analysis

Force signals were collected with transducer couplers (V-series, Coulbourn Instruments, Allentown, PA, USA). Analog-to-digital conversion was performed at 1 kHz with a 1401 plus A/D device and the data was recorded on computer using Spike2 software (Cambridge Electronic Design, Cambridge, UK). Analysis was performed manually off-line using the Spike2 software.

The peak force (N) of the trial with the greatest force was taken as the MVC force. The dependent outcomes from the constant force task were mean force of the non-detrended segment and the standard deviation (SD, N) and coefficient of variation (CV, SD/mean force × 100, %) of force for the same detrended segment. For the VIS/NOVIS combined trials, the force was maintained for 10 s for each vision condition. Similarly for both datasets, the epoch chosen for analysis was a 9 s segment that began 1 s after the target force was reached (VIS), or 1 s after the visual feedback was removed (NOVIS). For both Study 1 and 2, the data from all segments was detrended with the DC Remove function (1 s time constant) in Spike 2 (Tracy et al., 2007; Welsh et al., 2007; Jordan et al., 2013) to remove <0.5 Hz content (drift) from the signal. Importantly, this procedure removes the slow drift often observed for no-vision trials and preserves the force fluctuations of interest, allowing an appropriate comparison between segments from different vision conditions (see Figure 1). The SD of force is an absolute measure of the average variability during the segment. The CV of force (SD force/mean force × 100) is a normalized measure that expresses the absolute force variability as a fraction of the mean force exerted. It is the dependent outcome compared between subjects, who, due to strength differences, exert different absolute forces during the constant-force tasks.

### Statistical Analysis

Study 1: Repeated-measures analysis of variance (RMANOVA) was used. The between-subjects factors were age group (young, older) and sex (female, male). The within-subject factors were target force level (2.5, 30 and 65% MVC) and vision condition (VIS, NOVIS). The two test trials were averaged for a particular condition.

Study 2: For this single group of older adults, the dependent outcomes were compared between visual feedback conditions with a one-way RMANOVA. Two test trials were averaged to obtain the dependent outcome variables.

Combined analysis. The subject demographics, strength, CV of force, effect of visual feedback, and data analytic approach were similar between the older adult groups from Study 1 and Study 2. Therefore, the data from low force, high visual gain trials from both groups was pooled and the effects of visual feedback and age group effects were analyzed. A repeated measures ANOVA was used to examine the main effects of visual feedback between VIS and NOVIS and the age group × vision condition interaction. The within-subject factor was vision condition (VIS, NOVIS) and the between subjects factor was age group (young, older).

Bivariate Pearson correlations were used to quantify relations between variables. Exact *P*-values are provided where a single *P*-value conveys the result. Otherwise significance is denoted (*p* < 0.05, 0.01, 0.001) for clarity. Data in text is expressed as mean ± SD and in figures as mean ± standard error (SE).

## Results

### Subjects

Study 1: Height (1.71 ± 0.09 vs. 1.68 ± 0.11 m, *p* = 0.39) and body mass (72.8 ± 15.1 vs. 78.3 ± 15.6 kg, *p* = 0.29) were similar between young and older adults. Body mass index (BMI) tended to be lower for young compared with older adults (24.8 ± 4.38 vs. 27.5 ± 4.67 kg/m^2^, *p* = 0.07). The 16% reduction in MVC force for older vs. young adults was not statistically significant (34.3 ± 11.8 N vs. 41.0 ± 12.7 N, *p* = 0.11).

Study 2: For the single group of older adults, height was 1.65 ± 0.08m, body mass was 73.1 ± 12.7kg, and BMI was 26.8 ± 3.50 kg/m^2^. These values were not different from the older adults in Study 1 (*p* > 0.05).

### Force Control

#### Mean Force Exerted

Study 1: Young and older adults matched nominal constant force targets of 2.5, 30 and 65% of MVC force, with (VIS) and without (NOVIS) visual feedback (Figure 1). For the 2.5% MVC target, the mean force was similar for young and older adults (2.56 ± 0.22 vs. 2.68 ± 0.21% MVC, *p* = 0.11) for VIS segments. However, during NOVIS at 2.5% MVC, the mean force was greater than VIS for both young and old adults (*p* < 0.01) due to expected drift away from the target. For the NOVIS segment the mean force was significantly greater for older than young adults (3.53 ± 0.58 vs. 2.80 ± 0.52% MVC, *p* = 0.001).

At 30 and 65% target forces the older adults did not significantly alter their force output between VIS and NOVIS conditions. For older adults, the mean force was not different between VIS and NOVIS for the 30% MVC target force (30.9 ± 1.63 vs. 27.0 ± 3.20% MVC) and 65% MVC target force (63.6 ± 2.90 vs. 57.7 ± 6.72% MVC). However, for young adults the mean force was less during the NOVIS vs. VIS segment for both the 30% (29.6 ± 1.44–27.0 ± 3.20% MVC, *p* < 0.0001) and 65% (63.6 ± 2.92–57.7 ± 6.72% MVC, *p* < 0.0001) target forces.

Study 2: For these older adults the mean forces exerted were similar for VIS and NOVIS conditions (1.98 ± 0.69 vs. 2.14 ± 0.96% MVC, *p* = 0.39).

#### Standard Deviation (SD) of Force

Study 1: Pooled across vision conditions, the SD of force increased across target forces as expected (0.102 ± 0.007 N, 0.605 ± 0.053 N, 1.59 ± 0.115 N at 2.5, 30, and 65% MVC, respectively, *p* < 0.001). The increase in SD of force across target forces was greater for young compared with older adults (age group × target force interaction *p* < 0.05). Pooled across target forces, the SD of force was not different between VIS and NOVIS conditions (*p* = 0.82), and this effect was statistically similar between age groups (age group × target force interaction *p* = 0.88). For the 2.5% MVC target force, the SD of force was significantly greater for VIS than NOVIS (0.109 ± 0.007 N vs. 0.095 ± 0.007 N, *p* < 0.015).

Study 2: The SD of force was greater for VIS than NOVIS trials (0.047 ± 0.034 vs. 0.037 ± 0.034 N, *p* = 0.034).

#### Coefficient of Variation (CV) of Force

**Figure 2:**
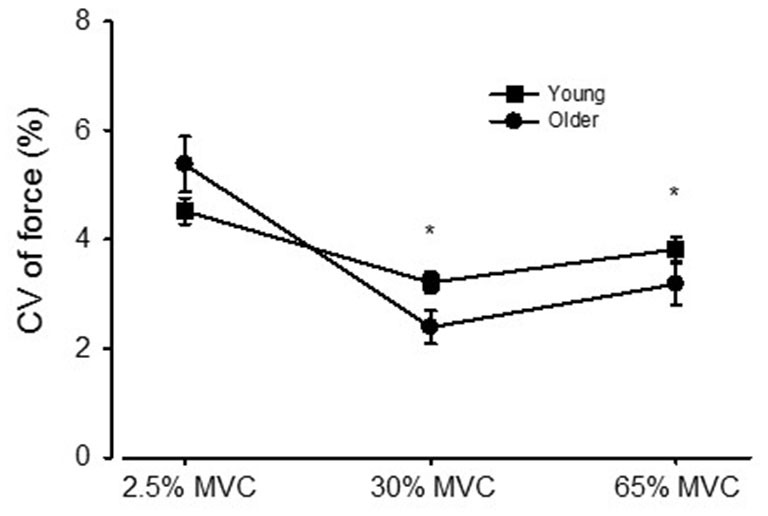
**The coefficient of variation (CV) of force for young and older adults across the 2.5, 30, and 65% MVC target forces, pooled across visual feedback conditions.** *p < 0.01 compared with the 2.5% MVC target force.

Study 1: Pooled across vision conditions, the CV of force declined from 4.94 ± 0.1.92% to 2.81 ± 1.48%, then increased to 3.49 ± 1.66% for the 2.5, 30, and 65% MVC target forces, respectively (*p* < 0.01 for each comparison, Figure 2). Pooled across target forces, there was an age group by vision condition interaction (*p* = 0.007) such that the increase in CV of force from NOVIS to VIS was significantly greater for older adults (3.26 ± 1.46% to 4.04 ± 1.38%) than young adults (3.88 ± 1.45% to 3.81 ± 1.40%).

**Figure 3:**
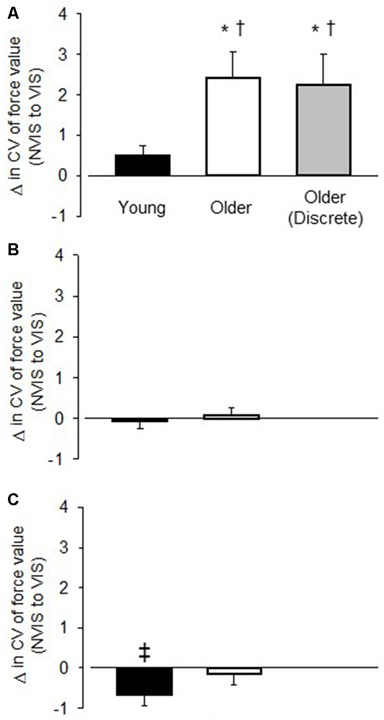
**The change in the CV of force value between the no visual feedback (NOVIS) and visual feedback (VIS) condition (VIS-NOVIS) for young adults (black bar), older adults (white bar), and older adults who performed discrete, randomly ordered test trials (gray bar). (A)** 2.5% MVC target force **(B)** 30% MVC target force, and **(C)** 65% MVC target force. Positive values indicate greater amplitude of fluctuations for VIS compared with NOVIS. Only **(A)** includes values for the older adults in Study 2 (gray bar), who performed low force, discrete, randomly ordered VIS and NOVIS trials. *p < 0.01 for VIS vs. NOVIS. ^†^*p* < 0.01 visual feedback effect greater for older adults than young adults. ^‡^*p* = 0.015 for VIS vs. NOVIS.

**Figure 4:**
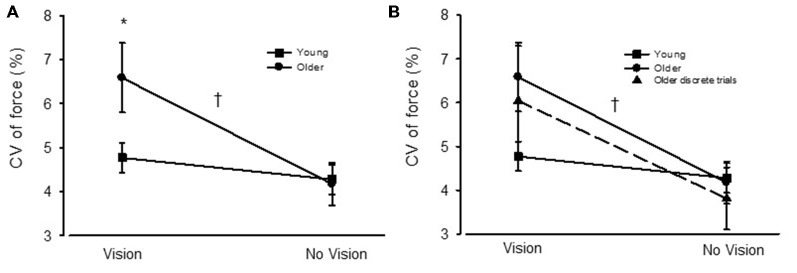
**(A)** The coefficient of variation (CV) of force for young (square symbols) and older adults (circles) during visual feedback (VIS) and no visual feedback (NOVIS) conditions. **(B)** The CV of force values for the older adults in Study 2 (discrete test trials) included in the figure (dashed line, triangles). *p < 0.05 for older adults vs. young adults. ^†^*p* = 0.002 for age group × visual condition interaction.

For young adults the CV of force was similar between VIS and NOVIS at both the 2.5% (4.77 ± 1.74 vs. 4.28 ± 1.78%) and 30% (3.18 ± 1.40 vs. 3.24 ± 1.28%) target forces but different at 65% MVC (3.49 ± 1.42 vs. 4.16 ± 1.61%, *p* = 0.015; Figures 3A–C). In contrast, for older adults the CV of force values were 58% greater for VIS than NOVIS at 2.5% MVC (6.59 ± 2.95% vs. 4.18 ± 1.80%, *p* = 0.003; Figures 3A, 4A). For older adults there was no difference between VIS and NOVIS at the 30% (2.42 ± 1.35 vs. 2.36 ± 1.82%) or 65% MVC (3.12 ± 1.99 vs. 3.26 ± 1.99%) target force levels (*p* > 0.05; Figures 3B,C). For the 2.5% MVC target force, the 58% increase in CV of force for older adults between NOVIS and VIS was greater than the non-significant 11% increase for young adults (age group × vision condition interaction *p* = 0.002; Figures 3A, 4A). There were no significant differences in visual feedback effects between men and women (sex × vision condition interaction *p* > 0.05).

Study 2: For the discrete, randomly ordered trials, the older adult group displayed a 59% greater CV of force value (*p* = 0.012) during VIS compared with NOVIS (6.04 ± 4.70 vs. 3.81 ± 2.6%, *p* = 0.012; Figure 4). The difference between visual feedback conditions was similar in magnitude to the similar low-force, high gain trials performed by the older adults in Study 1 (Figures 3, 4A).

### Combined Analysis

Due to the similar subject characteristics, similar CV of force values, similar differences in CV of force between VIS and NOVIS conditions, and similar analytic methods, we combined the two groups of older adults (Study 1, 2) for a more statistically powerful ancillary analysis of the CV of force values from the low-force, high visual gain trials during VIS and NOVIS. For the combined group of older adults (*N* = 28) the 58% greater CV of force values for VIS vs. NOVIS (6.31 ± 3.86 vs. 3.99 ± 2.23%, *p* < 0.0001) produced an observed statistical power of 0.995 and was similar to that observed for Study 1 and Study 2. For this combined analysis, this difference between VIS and NOVIS was significantly greater for the older adults than the young adults (4.77 ± 1.74 vs. 4.28 ± 1.78%, *p* = 0.063, *N* = 27). The age group × vision condition interaction was *p* = 0.002 and the observed power was 0.89.

## Discussion

**Figure 5:**
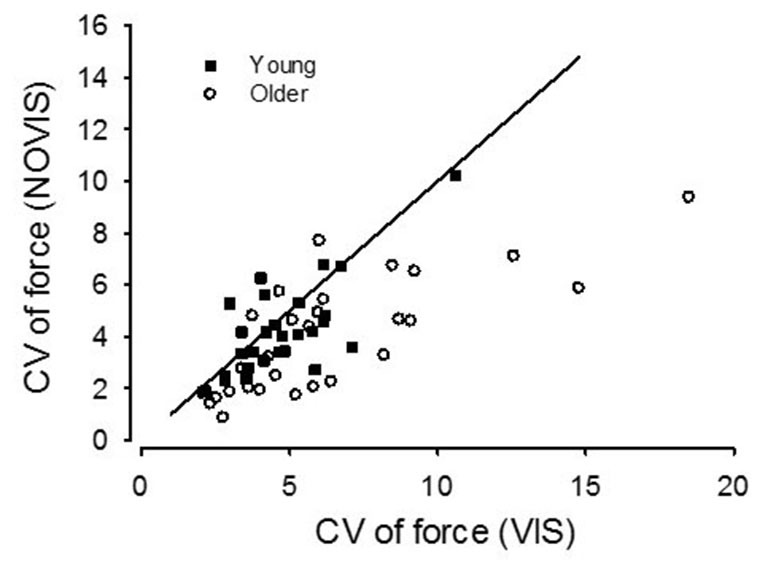
**Coefficient of variation (CV) values for the 2.5% MVC target force for young (square symbols) and older adults (circles).** The older adults from Study 2 are pooled with the older adults from Study 1. Visual feedback (VIS) values are plotted against no visual feedback (NOVIS) values. A line of identity is included (slope = 1.0). Symbols below the line indicate a subject with CV values greater for VIS than NOVIS.

The main findings were (1) for older adults performing low force contractions, high gain visual feedback increased the force fluctuations compared with no visual feedback; (2) this effect of visual feedback was significantly greater for older than young adults and was very consistent across the older adults (25/28 subjects, Figure 5); (3) only with high gain visual feedback at low forces were the older adults less steady than the young adults; and (4) during low-force high visual gain force tasks, the order of presentation of vision conditions had no effect on the contribution of visual processing to force fluctuations.

These findings agree with our similarly-designed studies in larger muscle groups such as the elbow flexors, knee extensors, ankle dorsiflexors, and ankle plantarflexors (Tracy et al., 2007; Welsh et al., 2007; Paxton et al., 2015). For example, in a study of the knee extensors that produced results very similar to the present work (Tracy et al., 2007), we showed that the normalized amplitude of force fluctuations during low force isometric contractions was 38% greater for older adults only when high gain visual force feedback was provided, compared with no visual feedback. Differences in force steadiness between young and older adults were erased during no-vision condition. From this same study the elbow flexor results were remarkably similar in that the CV of force was 36% greater for older adults during high gain visual feedback vs. no visual feedback, and there were no differences between young and older adults without visual feedback. Furthermore, in our other study of the knee extensors in a similarly-aged subject sample, there was a 38% increase in the CV of force for older adults but no change for young adults during visual feedback compared with no visual feedback (Welsh et al., 2007). Also, in our recent study of dorsiflexors and plantarflexors of healthy older adults (mean age 73 years) and older peripheral neuropathy patients (mean age 75 years), we observed significant 1.3-fold to 2-fold increases in the CV of force during low-force isometric contractions when high gain visual feedback was provided (Paxton et al., 2015). The changes in young adults (mean age 23 years) were not significant for the dorsiflexors (11% increase, *p* = 0.22) or relatively small for plantarflexors (28% increase, *p* = 0.05).

The present results also generally agree with findings from other groups on the greater visual feedback effects and apparent visuomotor processing impairment in older adults. For example, Sosnoff and Newell (2006a) found that older adults exhibited greater force fluctuations (1.9 fold greater) and longer estimated visuomotor processing times during isometric force control tasks where the intermittency of visual feedback was systematically manipulated across a 100-fold range. They also found that high visual gain exacerbated the force fluctuations during low force isometric contractions and that an index of information transmission was reduced for older adults, which suggested that limited information processing contributed to the impaired force control (Sosnoff and Newell, 2006b). The notion of longer processing times for older vs. young adults (160 vs. 150 ms) was also supported by two studies that varied intermittency of visual feedback and calculated estimated processing times (Slifkin et al., 2000; Vaillancourt et al., 2001). Without visual force feedback, Christou and Carlton (2001) failed to find differences in knee extensor force fluctuations between young and older adults (Christou and Carlton, 2001), but more recently Christou’s group clearly showed greater low-frequency force fluctuations in a hand muscles of older adults that was dependent on the gain of the visual force feedback (Kennedy and Christou, 2011; Fox et al., 2013; Baweja et al., 2015). Lastly, in a study of young and older adults, although Jordan et al did not observe greater CV of force values across increasing levels of visual gain, they did find a significant age group × visual gain interaction that appears to be driven by a difference in CV of force between no-vision and vision conditions (CV of force for vision ~6 vs. ~4% for no-vision; see their Figure 4B) that is strikingly similar to the present data (Jordan et al., 2013). Thus, in the context of (1) the significant accumulation of data from distal hand muscles; (2) the consistency of the visuomotor effect in our older adults (Figure 5); and (3) our similarly-executed work with larger proximal muscle groups and now with small distal hand muscles, it seems clear that the impaired force steadiness of older adults during low force isometric contractions is due to a reduced ability of older adults to employ visuomotor processing to correct force. In light of the preponderance of the direction of this literature, the previously observed age-related differences in isometric force steadiness (Galganski et al., 1993; Burnett et al., 2000; Laidlaw et al., 2000; Tracy and Enoka, 2002) can be confidently attributed to deficits in visuomotor processing. That this observation is true for multiple different effector muscle groups with widely varying characteristics (flexors and extensors, upper limb, lower limb, small distal muscles) further suggests a central processing-related phenomenon and probably not an effect of peripheral remodeling of motor unit pools with advanced age or other properties of the neural organization for that particular muscle. It is important to delimit this notion to isometric low force contractions, however. For example, the visuomotor processing-based mechanism can not necessarily be invoked to explain the greater difference in variability between young and older adults specifically during lengthening contractions, or the differences in variability observed between shortening and lengthening contractions (Laidlaw et al., 1999, 2000).

These robustness of these findings is further supported by observations beyond the strong age group × vision condition interaction we presented:

(1) For the older adults at 2.5% MVC, the force exerted was on average 32% greater for the NOVIS than VIS condition. This increase was greater for older than young adults (greater drift for older adults). Given the notion of signal dependent noise, the absolute fluctuations in force (SD of force) would be expected to be greater when the exerted force is greater (Hamilton et al., 2004). However, for older adults, despite a significant increase in the exerted force in the no-vision condition, the SD of force did not increase but was instead reduced. This observation speaks to the substantial reduction in the variability of the descending command to motor units and force fluctuations when older adults are not required to execute visuomotor processing during the task.
(2) In support of this notion, we previously observed a reduction in the variability of descending command upon removal of visual feedback, at least as was reflected in the less variable discharge behavior of single motor units in the knee extensors during low force isometric contractions (Welsh et al., 2007).
(3) Finally, because the older adults in Study 1 and Study 2 had similar characteristics, the experimental setup was similar, and the target forces for the low-force contractions were in the same low range with similarly high visual feedback gain, we combined the older adults from Study 2 with Study 1. With the larger and more evenly matched sample of 27 young and 28 older adults, the CV of force was 58% greater during VIS for older adults (observed power = 0.995) and the age group × visual condition interaction was *p* = 0.002, with an observed statistical power of 0.89. These results indicate a robust age-based difference in visuomotor correction.

The order of VIS/NOVIS presentation does not contribute to the effects. Our previous work in this area employed test trials where the subject completed a single ~20 s trial with a visual feedback segment followed by removal of visual feedback, always in that order. Our argument for this experimental strategy has been that the difference in the amplitude of the fluctuations and the clear qualitative change in the force traces immediately upon visual feedback removal suggested that the difference in CV of force between VIS and NOVIS was not likely due to an effect of order of presentation or due to a within-trial immediate learning effect. Also, our observation elsewhere (unpublished data) of no significant changes in the CV of force during lengthy, 30 s all-VIS trials suggested that the within trial effect was minimal. Furthermore, we found quite similar (58–59%) changes in the CV of force between VIS and NOVIS conditions for two separate samples of older adults, whether the trial was (1) a continuous VIS-NOVIS trial; or (2) the trials were discrete VIS and NOVIS trials in random order. The result effectively puts to rest the question of order effect or within-trial learning as a meaningful contributor to impaired isometric force steadiness in older adults.

The observation that muscle force output fluctuates despite volitional effort to minimize the variability, and that the amplitude of the fluctuations can change with human aging even in healthy older adults, is both physiologically and functionally interesting. Various groups have explored potential mechanisms that could explain impaired force steadiness in older adults, including peripheral motor unit changes (Galganski et al., 1993), altered antagonist muscle activity (Burnett et al., 2000), altered patterns of agonist muscle activation (Fox et al., 2013), greater motor unit discharge variability (Laidlaw et al., 2000; Welsh et al., 2007; Jordan et al., 2013), and altered central processing of sensory inflow and motor outflow (Sosnoff and Newell, 2006b). Ultimately, however, the changes in fluctuating behavior of either single motor units (Negro et al., 2009) or in the pattern of whole muscle activation (Fox et al., 2013) is a reflection of changes in the quality of the descending command of older adults during high gain, low force isometric contractions. The accumulating evidence now convincingly suggests a dominant contribution of impaired visuomotor processing which leads importantly to a more variable descending command, more variable motor unit activation, and greater force variability during low-force isometric contractions performed by older adults.

## Author Contributions

All authors (BT, LN, SW, RP, CF) substantially contributed to the work, drafted or revised intellectual content, approved of the final version, and agree to be accountable for the integrity and accuracy of the work.

## Conflict of Interest Statement

The authors declare that the research was conducted in the absence of any commercial or financial relationships that could be construed as a potential conflict of interest.
